# Genome-wide computational prediction of miRNAs in severe acute respiratory syndrome coronavirus 2 (SARS-CoV-2) revealed target genes involved in pulmonary vasculature and antiviral innate immunity

**DOI:** 10.22099/mbrc.2020.36507.1487

**Published:** 2020-06

**Authors:** Sandeep Saini, Avneet Saini, Chander Jyoti Thakur, Varinder Kumar, Rishabh Dilip Gupta, Jogesh Kumar Sharma

**Affiliations:** 1 Department of Bioinformatics, GGDSD College, Sector 32-C, 160030, Chandigarh, India; 2 Department of Biophysics, Panjab University, Sector 25, 160014, Chandigarh, India

**Keywords:** microRNA, coronavirus, target, COVID-19, pathways, gene silencing

## Abstract

The current outbreak of severe acute respiratory syndrome coronavirus 2 (SARS-CoV-2) in China threatened humankind worldwide. The coronaviruses contains the largest RNA genome among all other known RNA viruses, therefore the disease etiology can be understood by analyzing the genome sequence of SARS-CoV-2. In this study, we used an ab-intio based computational tool VMir to scan the complete genome of SARS-CoV-2 to predict pre-miRNAs. The potential pre-miRNAs were identified by ViralMir and mature miRNAs were recognized by Mature Bayes. Additionally, predicted mature miRNAs were analysed against human genome by miRDB server to retrieve target genes. Besides that we also retrieved GO (Gene Ontology) terms for pathways, functions and cellular components. We predicted 26 mature miRNAs from genome of SARS-CoV-2 that targets human genes involved in pathways like EGF receptor signaling, apoptosis signaling, VEGF signaling, FGF receptor signaling. Gene enrichment tool analysis and substantial literature evidences suggests role of genes like BMPR2 and p53 in pulmonary vasculature and antiviral innate immunity respectively. Our findings may help research community to understand virus pathogenesis.

## INTRODUCTION

The current outbreak of severe acute respiratory syndrome coronavirus 2 (SARS-CoV-2) was confirmed in 12,10956 peoples and there has been 67,594 deaths reported worldwide till now [1]. The genome sequence analysis of SARS-CoV-2 relates it to previously identified SARS-CoV (severe acute respiratory syndrome-related coronavirus, bat-SL-CoVZC45 and bat-SL-CoVZXC21) clade and hence it was classified under family *Coronaviridae* and genus *Betacoronavirus*. Furthermore, phylogenetic analysis suspected bat as the original host of the virus but possibility of intermediate host animal was also purposed [2-4]. 

A brief study of previously identified coronoviruses indicated six human coronaviruses (HCoVs) types: 229E, OC43, NL63, HKU1, SARS-CoV and Middle East respiratory syndrome (MERS-CoV). SARS-CoV and MERS-CoV are of zoonotic origin, and they have been outbreaks earlier during 2003 (China) and 2012 (Saudi Arabia) [5, 6]. The current disease clinical symptoms include fever, cough, dyspnoea which after chest radiography diagnosed as viral pneumonia, now named as COVID-19 (Coronavirus disease 2019) by WHO [7, 8]. The diagnostic determination of virus infection can be done by newly provided real-time RT-PCR assay [9].

MicroRNAs (miRNAs) are small (~22 nt) non-coding RNAs that play role in post-transcriptional gene regulation by binding to complementary sites on mRNA. The binding may results either in inhibition of translation or complete cleavage of mRNA depending upon complementarity of hybridization [10, 11]. The occurrence of miRNAs in plant, animal and fungi has been documented previously [12, 13]. Furthermore, the instances of viral encoded miRNAs in the host defense mechanism, cell differentiation, apoptosis and cell proliferation in different virus families and genus has been reported in literature [14]. 

The role of miRNAs in inducing the lung pathology, a characteristic symptom of SARS- CoV was identified previously by analyzing deep sequencing data from the lungs of SARS-CoV-MA15-infected BALB/c mice. 18-22 nucleotide long small viral RNAs were identified from genomic region of SARS-CoV, interestingly it was found that these small RNAs target the host cellular mRNAs 3’UTR specific target sequences and upon in vivo inhibition of these small viral RNAs by antisense inhibitor a significantly decrease in pulmonary inflammation was observed. [15]. Furthermore, the fact that existence of nuclear life cycle of SARS viruses exist was purposed by isolation of SARS-CoV from nucleus of Vero E6 Cells [16]. But the experimental approaches of miRNA identification was relied on expression in specific cell type and therefore based on time consuming cloning techniques. In the urgent need to understand the disease etiology, the computational based miRNAs prediction approaches can provide early evidences by genome analysis to predict miRNAs in timely manner, so that the impact of miRNAs on disease etiology can be traced during outbreaks or emergence [17, 18]. 

Mainly two approaches have been used for computational miRNAs prediction: ab-intio based and homology based. Homology based approach depends on evolutionary conservation, and therefore limited in locating novel miRNAs in genome. Whereas ab-intio based approach scan for hair-pin loop fold in genome to detect novel pre-miRNAs therefore is more significant [19, 20]. 

Till date, the RNA viruses-encoded miRNAs have been predicted in Hepatitis-A virus (HAV), Hepatitis-E virus (HEV), Dengue virus (DENV), ZIKA Virus ZIKV), Ebola virus, Japanese Encephalitis virus (JEV), Kyasanur forest disease virus (KFDV) and Nipah virus [21-28]. Therefore, in this study the genome of SARS-CoV-2 was being analysed to predict mature viral miRNAs. Moreover, predicted mature viral miRNAs were also scanned for target genes in human genome and these targets were further analysed for gene ontology. 

## MATERIALS AND METHODS

**Genome Data Retrieval & Analysis:** The complete genome sequence of SARS-CoV-2 was retrieved from NCBI genome database (https://www.ncbi.nlm.nih.gov/genome/) using accession number: MN908947. Genome is positive sense single stranded RNA molecule with linear topology. It contains 29903 nucleotide (nt) base pair.

An ab-intio based pre-miRNA prediction software package, Vmir (v2.3) was used for identification of SARS-CoV-2’s pre-miRNAs. VMir package contains two individual modules: VMir analyzer and VMir viewer for prediction and viewing pre-miRNAs respectively [29]. The analysis was done using default parameters (window count: 500, conformation: linear, orientation: both) in VMir analyzer. Furthermore, filtering parameter (min. hairpin size: 70, min. score: ≥150 and min. window count: ≥35) was used in VMir viewer to filter out top scorer pre-miRNAs as described previously in literature [21, 25]. 

**Identification of Potential pre-miRNAs & mature miRNAs: **Potential pre-miRNAs were identified by using ViralMir (http://csb.cse.yzu.edu.tw/viralmir/), a SVM (support vector machine) based web-server. ViralMir was specially designed for viruses with SVM model for prediction has been trained on sequence and structural features of experimentally validated pre-miRNAs data set [30]. The Mfold (http://unafold.rna.albany.edu/?q=mfold) web server was used with default parameters to predict the secondary structure **(**Supplementary Fig. S1.**)** and minimum free energy (MFE) of pre-miRNAs [31]. Mature Bayes (http://mirna.imbb.forth.gr/MatureBayes.html) web-server was used for identification of mature miRNAs from filtered pre-miRNAs. Mature Bayes uses Naive Bayes Classifier (NBC) and takes into account sequence as well as structural information of experimental predicted miRNA precursors for deducing mature miRNAs from precursors [32]. 

**Prediction of target genes & GO (Gene Ontology) analysis: **Target prediction of mature miRNAs against human genome was done using in an online web based server, miRDB (http://mirdb.org/). The custom prediction module of server was used for predicting target genes in human. The server uses seeding approach and scans viral mature miRNAs against 3’ UTR (untranslated regions) of human’s genome for possible hybridization [33]. GO analysis of the target genes was performed using PANTHER (Protein Analysis through Evolutionary Relationships) (http:// www.pantherdb.org) and Enrichr (https://amp.pharm.mssm.edu/Enrichr/) to explore the role of target gene’s product in biological process, molecular function, cellular component and pathways [34, 35]. NCBI’s Gene IDs were used for this analysis to find GO terms related to gene products. The associations of screened target genes with related pathways were established by literature evidences to deduce disease etiology.

## RESULTS

VMir analysis predicted a total of 1114 hair-pin like pre-miRNAs folds in SARS-CoV-2 genome that were filtered using filtering parameter as described in methodology above. After filtering done by VMir viewer only top 13 pre-miRNAs were selected for further study. Nine pre-miRNAs were found on direct strand whereas four pre-miRNAs on reverse strand. Additionally, all 13 pre-miRNAs were in length range 78-148 nt. The sequence, rank, score, length and orientation are listed in Table 1. 

As ab-intio based tools have the limitation of false-positive pre-miRNAs prediction because of selection of the pseudo hair pin loops structures [36, 37] therefore to validate and find reliable candidates pre-miRNAs, all 13 predicted pre-miRNAs were further analysed by ViralmiR for identification for real or pseudo viral pre-miRNAs. All 13 pre-miRNAs were found to be real or potential pre-miRNAs folds, which were further confirmed by assessment of minimum free energy (MFE) by Mfold Table 2. Because pre-miRNAs sequence folding is one of the feature that confer stability to structural fold therefore by calculating MFE more confidence in authenticity of predicted pre-miRNAs can be done [38]. 

After authentication of pre-miRNAs, Mature Bayes server was used for retrieving the mature miRNAs. A total of 26 mature miRNAs were obtained from 13 precursors on 5’ and 3’ stem location as shown in Table 3. As mentioned in literature, one or both strands can serve as mature miRNA molecule depending on the assembly of RISC complex therefore we retained both for further analysis [39]. 

**Table 1:** pre-miRNAs rank, orientation, size, genomic position, score and sequence as predicted by VMir

**S.** ** No**	**Predicted pre-miRNAs**	**Rank**	**Orientation**	**Size (nt)**	**Genomic Position**	**VMir Score**	**pre-miRNAs Sequence**
1	SARS-CoV-2-MD135	1	Direct	101	11234-11334	211.4	GCUAGUUGGGUGAUGCGUAUUAUGACAUGGUUGGAUAUGGUUGAUACUAGUUUGUCUGGUUUUAAGCUAAAAGACUGUGUUAUGUAUGCAUCAGCUGUAGU
2	SARS-CoV-2-MD241	2	Direct	110	21131-21240	204.7	AACAAAAGCUAGCUCUUGGAGGUUCCGUGGCUAUAAAGAUAACAGAACAUUCUUGGAAUGCUGAUCUUUAUAAGCUCAUGGGACACUUCGCAUGGUGGACAGCCUUUGUU
3	SARS-CoV-2-MR243	3	Reverse	148	23563-23710	188.8	UGUGGGUAUGGCAAUAGAGUUAUUAGAGUAAGCAACUGAAUUUUCUGCACCAAGUGACAUAGUGUAGGCAAUGAUGGAUUGACUAGCUACACUACGUGCCCGCCGAGGAGAAUUAGUCUGAGUCUGAUAACUAGCGCAUAUACCUGCA
4	SARS-CoV-2-MR304	4	Reverse	94	29532-29625	184.6	GAAUUCAUUCUGCACAAGAGUAGACUAUAUAUCGUAAACGGAAAAGCGAAAACGUUUAUAUAGCCCAUCUGCCUUGUGUGGUCUGCAUGAGUUU
5	SARS-CoV-2-MR155	5	Reverse	109	14973-15081	183.9	GGCAUACUUAAGAUUCAUUUGAGUUAUAGUAGGGAUGACAUUACGUUUUGUAUAUGCGAAAAGUGCAUCUUGAUCCUCAUAACUCAUUGAAUCAUAAUAAAGUCUAGCC
6	SARS-CoV-2-MD20	6	Direct	107	2107-2213	180.1	UAACAUCUUUGGCACUGUUUAUGAAAAACUCAAACCCGUCCUUGAUUGGCUUGAAGAGAAGUUUAAGGAAGGUGUAGAGUUUCUUAGAGACGGUUGGGAAAUUGUUA
7	SARS-CoV-2-MD110	7	Direct	128	9797-9924	179.1	GCUGCGCUGUGCACCUUUUUGUUAAAUAAAGAAAUGUAUCUAAAGUUGCGUAGUGAUGUGCUAUUACCUCUUACGCAAUAUAAUAGAUACUUAGCUCUUUAUAAUAAGUACAAGUAUUUUAGUGGAGC
8	SARS-CoV-2-MD3	8	Direct	94	398-491	171.8	CAUCUUAAAGAUGGCACUUGUGGCUUAGUAGAAGUUGAAAAAGGCGUUUUGCCUCAACUUGAACAGCCCUAUGUGUUCAUCAAACGUUCGGAUG
9	SARS-CoV-2-MD85	9	Direct	78	8144-8221	170.2	GACAAUGUCUUAUCUACUUUUAUUUCAGCAGCUCGGCAAGGGUUUGUUGAUUCAGAUGUAGAAACUAAAGAUGUUGUU
10	SARS-CoV-2-MD308	10	Direct	119	26995-27113	169.1	GCUGUGACAUCAAGGACCUGCCUAAAGAAAUCACUGUUGCUACAUCACGAACGCUUUCUUAUUACAAAUUGGGAGCUUCGCAGCGUGUAGCAGGUGACUCAGGUUUUGCUGCAUACAGU
11	SARS-CoV-2-MD229	11	Direct	80	20002-20081	159.3	GAUGGUCAAGUAGACUUAUUUAGAAAUGCCCGUAAUGGUGUUCUUAUUACAGAAGGUAGUGUUAAAGGUUUACAACCAUC
12	SARS-CoV-2-MD30	12	Direct	131	2981-3111	152.8	UACUUAUUUGAUGAGUCUGGUGAGUUUAAAUUGGCUUCACAUAUGUAUUGUUCUUUCUACCCUCCAGAUGAGGAUGAAGAAGAAGGUGAUUGUGAAGAAGAAGAGUUUGAGCCAUCAACUCAAUAUGAGUA
13	SARS-CoV-2-MR186	13	Reverse	101	18089-18189	152.7	GGUCAUGUCCUUAGGUAUGCCAGGUAUGUCAACACAUAAACCUUCAGUUUUGAAUUUAGUGUCAACACUGAGGUGUGUAGGUGCCUGUGUAGGAUGUAACC

**Table 2:** Potential pre-miRNAs as validated by ViralMir and their Minimum Free Energy (MFE) calculated by Mfold

**Predicted pre-miRNA**	**Potential**	**Minimum Free Energy (MFE) (-∆G.kcal/mol)**
SARS-CoV-2-MD135	Potential	-36.20
SARS-CoV-2-MD241	Potential	-38.10
SARS-CoV-2-MR243	Potential	-44.00
SARS-CoV-2-MR304	Potential	-28.30
SARS-CoV-2-MR155	Potential	-28.80
SARS-CoV-2-MD20	Potential	-29.30
SARS-CoV-2-MD110	Potential	-35.00
SARS-CoV-2-MD3	Potential	-25.50
SARS-CoV-2-MD85	Potential	-23.80
SARS-CoV-2-MD308	Potential	-36.60
SARS-CoV-2-MD229	Potential	-21.30
SARS-CoV-2-MD30	Potential	-40.30
SARS-CoV-2-MR186	Potential	-32.50

**Table 3:** Mature miRNAs length, location and sequence as predicted by MatureBayes

**Mature miRNAs**	**Length (nt)**	**Location**	**Mature miRNAs sequence**
SARS-CoV-2-MD135 5P	22	5'	AUACUAGUUUGUCUGGUUUUAA
SARS-CoV-2-MD135 3P	22	3'	UGUGUUAUGUAUGCAUCAGCUG
SARS-CoV-2-MD241 5P	22	5'	UUGGAGGUUCCGUGGCUAUAAA
SARS-CoV-2-MD241 3P	22	3'	UGAUCUUUAUAAGCUCAUGGGA
SARS-CoV-2-MR243 5P	22	5'	GGCAAUGAUGGAUUGACUAGCU
SARS-CoV-2-MR243 3P	22	3'	UGAUAACUAGCGCAUAUACCUG
SARS-CoV-2-MR304 5P	22	5'	AAGAGUAGACUAUAUAUCGUAA
SARS-CoV-2-MR304 3P	22	3'	UUUAUAUAGCCCAUCUGCCUUG
SARS-CoV-2-MR155 5P	22	5'	CAUUUGAGUUAUAGUAGGGAUG
SARS-CoV-2-MR155 3P	22	3'	AAAAGUGCAUCUUGAUCCUCAU
SARS-CoV-2-MD20 5P	22	5'	ACUCAAACCCGUCCUUGAUUGG
SARS-CoV-2-MD20 3P	22	3'	UAAGGAAGGUGUAGAGUUUCUU
SARS-CoV-2-MD110 5P	22	5'	UGAUGUGCUAUUACCUCUUACG
SARS-CoV-2-MD110 3P	22	3'	CUUACGCAAUAUAAUAGAUACU
SARS-CoV-2-MD3 5P	22	5'	AAAAGGCGUUUUGCCUCAACUU
SARS-CoV-2-MD3 3P	22	3'	ACUUGAACAGCCCUAUGUGUUC
SARS-CoV-2-MD85 5P	22	5'	UUAUCUACUUUUAUUUCAGCAG
SARS-CoV-2-MD85 3P	22	3'	UUGUUGAUUCAGAUGUAGAAAC
SARS-CoV-2-MD308 5P	22	5'	UGCUACAUCACGAACGCUUUCU
SARS-CoV-2-MD308 3P	22	3'	GAGCUUCGCAGCGUGUAGCAGG
SARS-CoV-2-MD229 5P	22	5'	UAAUGGUGUUCUUAUUACAGAA
SARS-CoV-2-MD229 3P	22	3'	AGUGUUAAAGGUUUACAACCAU
SARS-CoV-2-MD30 5P	22	5'	CUGGUGAGUUUAAAUUGGCUUC
SARS-CoV-2-MD30 3P	22	3'	UUUGAGCCAUCAACUCAAUAUG
SARS-CoV-2-MR186 5P	22	5'	AGUUUUGAAUUUAGUGUCAACA
SARS-CoV-2-MR186 3P	22	3'	ACUGAGGUGUGUAGGUGCCUGU

Computational prediction of miRNA-mRNA binding depends on Watson-Crick base pairing which is mostly implemented using seed pairing approach [40]. miRDB also adopts 7-mer seeding approach through MirTarget algorithm. We predicted 1059 human target genes (Supplementary Table S1**)** by custom prediction using mature miRNAs sequences which bind at 3’ UTRs. We selected top scoring target genes with prediction score >80 because score above this threshold are most likely to be real and not required any other supporting evidence [33]. 

**Figure 1:**
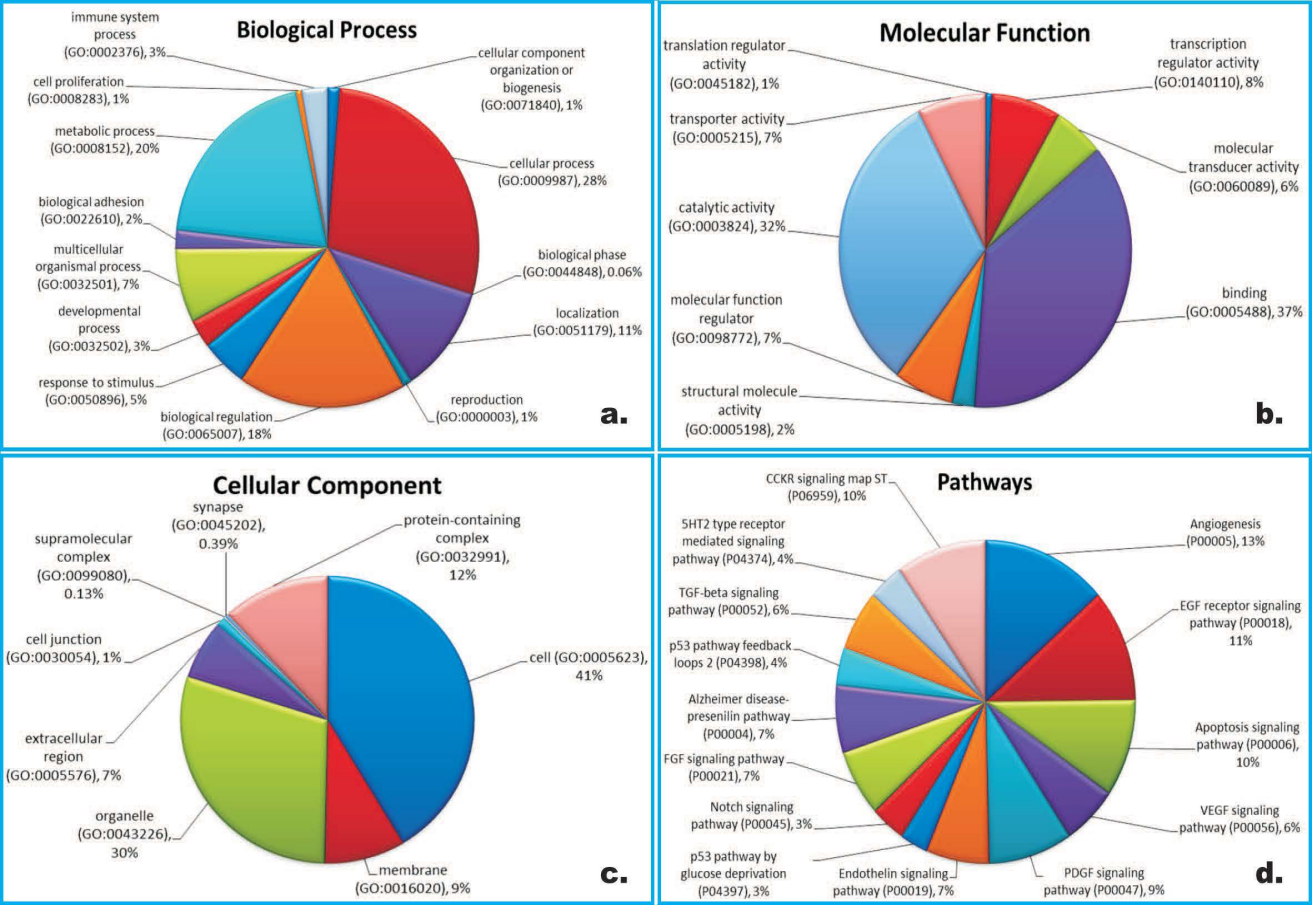
Gene Ontology analysis by PANTHER & Enrichr. The GO terms retrieved by PANTHER database clustered predicted target genes based on biological process (a), molecular function (b) and cellular component (c). The pathways enrichment analysis was done by Enrichr and only significant pathways with p value <0.1 are listed (d)

Gene Ontology term for the target genes were identified by PANTHER database which cluster and group them into biological process (Fig. 1a), molecular function (Fig. 1b) and cellular component (Fig. 1c). Biological processes important for antiviral responses are immune system process (GO:0002376), metabolic process (GO:0008152), biological adhesion (GO:0022610), biological regulation (GO:0065007) and response to stimulus (GO:0050896). Molecular functions were classified into eight categories essentially comprised of transporter activity (GO:0005215), catalytic activity (GO:0003824) translation regulator activity (GO:0045182), transcription regulator activity (GO:0140110) and binding (GO:0005488). Cellular Components encompasses eight subcellular components including extracellular region (GO:0005576), cell (GO:0005623) and organelle (GO:0043226).

Target genes were further evaluated using Enrichr, a gene list enrichment analysis tool which retrieved total 82 pathways. On the basis of p value<0.1 few important pathways associated with targeted gene are listed in (Fig. 1d) which are important in human immune response to virus infection including angiogenesis (P000050), EGF receptor signaling pathway (P000180), apoptosis signaling pathway (P000060), VEGF signaling pathway (P000560), FGF receptor signaling pathway (P00021) and CCKR signaling map ST pathway (P06959). Screened target genes and their associated roles that may involve in virus pathogenesis were listed in Table 4. along with literature evidence.

**Table 4:** Screened target genes and their associated role with literature evidences

**Mature miRNAs**	**Gene**	**Gene Description**	**Role**	**PubMed ID**
SARS-CoV-2-MD241-3P	BMPR2	bone morphogenetic protein receptor type 2	Pulmonary vasculature	30149506
SARS-CoV-2-MD3 -3P	p53	Tumor suppressor p53	Antiviral innate immunity	21994612, 22978174

## DISCUSSION

In the genomic-age, there are now more ways to find and studying miRNA biology, the most trending one is the genome-wide identification of this small non-coding RNAs [41]. The ab-intio prediction approach even can detect miRNAs that were not identified in cloning experiments due to under expression [42]. There are now ample amount of evidences that several viruses encode miRNAs, which directly downregulate the expression of genes involve in immunological, apoptosis, axon guidance and cell differentiation pathways [43, 44]. 

The significance of miRNAs in viral induced respiratory infection and immune regulation has been established previously [15]. Here in this study, the genome analysis of recent outbreak SARS-CoV-2 was done to explore the role of miRNAs in acute respiratory syndrome. 

We found significant pathways that may contribute to disease etiology for example apoptosis play an important role in physiological processes and pathogenesis of infectious diseases caused by viruses. nCoV-MD3 -3P target p53 which act as a main inducer of apoptosis pathway during viral infection [45]. p53-dependent apoptosis has been reported to control viral infection of herpes simplex virus (HSV), vesicular stomatitis virus (VSV) and polio virus [46]. Tumor suppressor p53 (TP53) diminish the ability of viral replication and spreading as well as up regulate many genes of type I IFN transcriptional target that suggest p53 role in innate immunity [47].

nCoV-MD241-3P target BMPR2 (bone morphogenetic protein receptor type 2) which involved in transforming growth factor (TGF)-β signaling pathway. Upon viral infection, BMPR2 gets suppressed which result inhibition of pulmonary vascular homeostasis [48]. 

The previous studies on different viral miRNAs and their target gene silencing explained interesting facts, particularly about disease etiology [49-51]. Above all, the occurrence of same miRNAs in in-vitro studies as is found by computational means build confidence in in-silico approaches [52]. To our knowledge this is the first paper on computational prediction of mature miRNAs from SARS-CoV-2 genome where we found that predicted mature miRNAs are targeting the large number of significant human target genes. The main findings of the work are the two target genes: BMPR2 and TP53 that involves in the pulmonary vasculature and antiviral innate immunity respectively. The inhibition of these two target genes by predicted viral miRNAs may induce the respiratory lung disease pathology and decrease in antiviral response of the body. This study may results in exploring the disease manifestation.
